# Interleukin-1 Receptor Antagonist Protects against Lipopolysaccharide Induced Diaphragm Weakness in Preterm Lambs

**DOI:** 10.1371/journal.pone.0124390

**Published:** 2015-04-10

**Authors:** Kanakeswary Karisnan, Anthony J. Bakker, Yong Song, Peter B. Noble, J. Jane Pillow, Gavin J. Pinniger

**Affiliations:** 1 School of Anatomy, Physiology and Human Biology, University of Western Australia, Crawley, WA, Australia; 2 Centre for Neonatal Research and Education, School of Paediatrics and Child Health, University of Western Australia, Crawley, WA, Australia; University of Louisville School of Medicine, UNITED STATES

## Abstract

Chorioamnionitis (inflammation of the fetal membranes) is strongly associated with preterm birth and *in utero* exposure to inflammation significantly impairs contractile function in the preterm lamb diaphragm. The fetal inflammatory response to intra-amniotic (IA) lipopolysaccharide (LPS) is orchestrated via interleukin 1 (IL-1). We aimed to determine if LPS induced contractile dysfunction in the preterm diaphragm is mediated via the IL-1 pathway. Pregnant ewes received IA injections of recombinant human IL-1 receptor antagonist (rhIL-1ra) (Anakinra; 100 mg) or saline (Sal) 3 h prior to second IA injections of LPS (4 mg) or Sal at 119d gestational age (GA). Preterm lambs were killed after delivery at 121d GA (term = 150 d). Muscle fibres dissected from the right hemi-diaphragm were mounted in an *in vitro* muscle test system for assessment of contractile function. The left hemi-diaphragm was snap frozen for molecular and biochemical analyses. Maximum specific force in lambs exposed to IA LPS (Sal/LPS group) was 25% lower than in control lambs (Sal/Sal group; p=0.025). LPS-induced diaphragm weakness was associated with higher plasma IL-6 protein, diaphragm IL-1β mRNA and oxidised glutathione levels. Pre-treatment with rhIL-1ra (rhIL-1ra/LPS) ameliorated the LPS-induced diaphragm weakness and blocked systemic and local inflammatory responses, but did not prevent the rise in oxidised glutathione. These findings indicate that LPS induced diaphragm dysfunction is mediated via IL-1 and occurs independently of oxidative stress. Therefore, the IL-1 pathway represents a potential therapeutic target in the management of impaired diaphragm function in preterm infants.

## Introduction

A functioning diaphragm is critically important for the initiation and sustainment of spontaneous unsupported breathing. However, like the lung, the preterm diaphragm is structurally and functionally immature at birth and therefore is poorly equipped to meet the mechanical demands of breathing. Inadequate diaphragm development may also enhance the vulnerability to additional *in utero* exposures that are strongly associated with very preterm births such as chorioamnionitis. IL-1 has a critical role in the inflammatory pathway associated with pulmonary responses to chorioamnionitis [1]. Identification of a similar pathway as a determinant of an adverse impact of antenatal inflammation on diaphragm function would support treatment strategies targeting inhibition of the IL-1 pathway.

The preterm diaphragm needs to generate sufficient inspiratory force to overcome the mechanical disadvantages imposed by a highly compliant chest wall, low levels of endogenous surfactant and noncompliant, structurally immature lungs. However, preterm infants have significantly lower twitch trans-diaphragmatic pressure [2], a low proportion of type I and a high proportion of immature, neonatal muscle fibres compared to term infants [3,4]. Furthermore, the maximum force producing capacity of the diaphragm in preterm sheep is significantly lower than the term counterparts [5]. These characteristics suggest functional impairment of the diaphragm that may impede adequate ventilation. Additionally, as weak muscles need to work closer to maximum contractile capacity, preterm infants may be predisposed to the development of respiratory muscle fatigue, thereby contributing to postnatal respiratory failure.

Data from a number of species indicate that the immature diaphragm contains a low proportion (<10%) of type I fatigue resistant muscle fibres, compared to the adult diaphragm (50–60% type I fibres) [3,4,6]. Although this suggests the preterm diaphragm is highly susceptible to fatigue, numerous studies report the opposite finding of a high fatigue resistance in the diaphragm of newborn rats [7] cats [8] and baboons [4]. For the most part, these studies have evaluated muscle fatigue under *in vitro* conditions using repeated isometric contraction of isolated diaphragm tissue. The fatigue resistance of the immature diaphragm under *in situ* conditions is less widely studied, but may differ from the *in vitro* condition [9]. Maxwell *et al* reported that primate immature diaphragm has high proportion of immature muscle fibres that are highly oxidative and high mitochondrial content that may contribute to fatigue resistance [4]. These findings suggest that the relationship between MHC expression, contractile function and fatigability is less robust in fetal muscle compared to adult muscle.

In addition to immaturity, antenatal inflammation may further exacerbate diaphragm dysfunction in preterm infants. About 70% of preterm births are associated with intra-uterine infection which commonly manifests as chorioamnionitis [10]. Chorioamnionitis frequently induces a systemic fetal inflammatory response syndrome (FIRS) causing multiple organ injury and adverse neonatal outcomes [11]. FIRS is mediated by pro-inflammatory cytokines (IL-1, IL-6 and TNF-α) and diagnosed clinically by increased plasma IL-6 levels and funisitis [10,12]. Increased cytokine secretion in inflammatory diseases is commonly linked with the development of muscle weakness [13]. Circulating pro-inflammatory cytokines play an important role in diaphragm weakness in mice after exposure to intraperitoneal lipopolysaccharide (LPS) [14]. Pro-inflammatory cytokines may reduce force production directly through disruption to Ca^2+^ handling or altered sensitivity of myofilaments to Ca^2+^, or indirectly via myofibre atrophy or increased production of reactive oxygen species (ROS) [15,16,17]. Developing diaphragm myofibres contain large numbers of mitochondria [4] and have a less efficient antioxidant defence system [18], suggesting that the preterm diaphragm is also prone to oxidative stress. Increased mitochondrial production of ROS may contribute to muscle weakness via activation of proteolytic pathways and myofibre atrophy [13] or by altering excitation-contraction coupling.

Previously we showed that a two day intra-amniotic (IA) exposure to LPS causes diaphragm weakness in preterm lambs and is associated with mitochondrial oxidative stress and electron chain dysfunction [19]. However, IA LPS exposure also increases IL-10β expression in the diaphragm and increases systemic IL-6 protein [20]. Importantly, IL-1 signalling plays a key role in IA LPS induced lung and systemic inflammation in fetal lambs [1,21]. Blockade of IL-1 signalling in the amniotic cavity using rhIL-1ra inhibits both lung and systemic inflammatory responses [1]. It is unknown whether IA LPS induced diaphragm weakness is mediated primarily via IL-1 signalling or is associated with oxidative stress.

This study investigates the role of IL-1 signalling and oxidative stress on IA LPS induced diaphragm weakness in preterm lambs. We hypothesised that blockade of IL-1 signalling ameliorates diaphragm dysfunction induced by IA LPS exposure.

## Methods

### Animals and Experimental Design

All experiments were conducted in accordance with the guidelines of the National Health and Medical Research Council Code of practice for the care and use of animals for scientific purposes and were approved by the University of Western Australia Animal Ethics Committee (Approval Number: 3/400/1023). Pregnant Merino ewes were randomised to ultrasound guided IA rhIL-1ra (100 mg; Kineret (Anakinra); Amgen, CA, USA) or saline (Sal) injections 3 h prior to a second IA injection of LPS (4 mg; *Escherichia coli* 055:B5, Sigma Chemical, St. Louis, MO) or Sal at 119 d gestational age (GA) generating two experimental groups (Sal/LPS, n = 7; rhIL-1ra/LPS, n = 8) and two control groups (Sal/Sal, n = 7; rhIL-1ra/Sal, n = 8). Preterm lambs were delivered at 121 d GA (term = 150 d) via caesarean section and killed immediately with pentobarbitone (150 mg/kg IV, Pitman-Moore, NSW, Australia). Longitudinal muscle fibre strips were dissected from the right hemi-diaphragm and used for assessment of contractile function. The left hemi-diaphragm was immediately snap frozen in liquid nitrogen for molecular and biochemical analyses. Blood samples collected from the umbilical artery were centrifuged (3 000 RPM, 10 min, 4°C): the systemic response to IA LPS exposure was determined from the plasma supernatant. All samples were snap frozen in liquid nitrogen and stored at -80°C prior to analysis.

### Diaphragm contractile function

Contractile measurements were performed according to Song et al [20]. A longitudinal strip of diaphragm muscle fibres (3–5 mm wide) was isolated with a portion of the central tendon at one end and rib attachment on the other end. The ends were tied with surgical silk thread and the preparation was mounted in an *in vitro* muscle test system (model 1205, Aurora Scientific In., Aurora, Canada) containing Krebs physiological salt solution (in mM: NaCl, 109; KCl, 5; MgCl2, 1; CaCl2, 4; NaHCO3, 24; NaH2PO4, 1; sodium pyruvate, 10). The organ bath was maintained at 25°C and continuously bubbled with 95% O_2_ /5% CO_2_.

The muscle strip was manually adjusted to the optimal muscle length (L_0_) upon which maximum isometric twitch force (P_t_) was recorded. L_0_ was measured using a digital calliper. Time to peak (TTP), half relaxation time (1/2 RT) and maximum rate of force development (df/dt) of twitch contractions were determined using the DMA software (Aurora Scientific In., Aurora, Canada). The fatigue resistance of the diaphragm was assessed by a series of 150 tetanic contractions (300 ms contraction times at 60 Hz once every second). The fatigue index (FI) was determined from the ratio of the force produced during the 150^th^ contraction relative to the 1^st^ contraction[6], in which a higher number indicates a greater fatigue resistance. P_0_ and P_t_ were normalised for cross-sectional area (CSA) and expressed as specific force (N/cm^2^). CSA was estimated as muscle mass (g) / (L_0_ x muscle density (1.056 g/cm^3^)).

### Muscle protein extraction

Total protein extraction of the diaphragm and analysis of protein concentration were performed as described previously [20].

### IL-1β and IL-6 plasma levels

Plasma IL-1β and IL-6 protein concentrations were measured using a sandwich ELISA assay [20]. The wells in 96-well microplate (High binding, Microlon Greiner Bio-One, Frickenhausen, Germany) were coated with 100 μL of capture antibodies from SeroTec (5 μg/mL; MCA1658 for IL-1β and MCA1659 for IL-6, East Brisbane, Australia) in 0.1 M carbonate buffer (pH 9.6) at 4°C overnight. The wells were blocked with 3% skim-milk solution in phosphate buffered saline (PBS: pH 7.2) for 1 h, then washed three times with PBS containing 0.05% Tween 20 (PBST). Plasma samples were added and incubated for 2 hours at room temperature. After washing three times with PBST, the detection antibodies from SeroTec (2 μg/mL; AHP423 for IL-1β and AHP424 for IL-6, East Brisbane, Australia) were added into the wells and incubated for 2 hours at room temperature. The wells were subsequently washed as above and the bound antigen was detected with goat anti-rabbit IgG-HRP (1:2000; 7074S Cell Signalling Technology, Carlsbad CA, USA). Colour development was initiated by adding 3,3’,5,5’-tetramethyl-benzidine liquid substrate (Sigma, Castle Hill, Australia) and was stopped after 15 min by adding 0.5 M sulphuric acid. The optical density (OD) was measured at 450 nm on a microplate reader (Labtec Multiskan, Wals, Austria).

### Myeloperoxidase (MPO) staining

To quantify intra-muscular neutrophil infiltration, diaphragm sections (8 μm thickness) were incubated with anti-myeloperoxidase polyclonal antibody (CMC28917023, Cell Marque, CA, USA) 1:100 dilution overnight at 4°C. Preterm lamb liver sections were used as positive controls. VECTASTAIN ELITE ABC kit (PK-6200, Vector Laboratories, Burlingame, CA, USA) and ImmPACT 3, 3’-diaminobenzidine (DAB) peroxidase substrate (SK-4105, brown, Vector Laboratories, Burlingame, CA, USA) were used to identify the MPO positive cells. Sections were counterstained with haematoxylin for 45 seconds and cover slip applied using VectaMount AQ mounting medium (Vector Laboratories, Burlingame, CA, USA). Sections were imaged using a light microscope (Nikon, NY, USA) at 400X magnification.

### MPO assay

Analysis of diaphragm MPO activity was obtained from total protein extract: 50 μL protein extract was pipetted into microplate wells and 200 μL phosphate buffer (pH 6.0 containing 0.167 mg/mL O-dianisidine dihydrochloride and 0.0005% hydrogen peroxide) were added to each sample well. Lysis buffer for protein extraction was used for negative control wells. After three minutes incubation, the optical density was measured at 450 nm using a microplate reader (Labtec Multiskan, Wals, Austria). MPO activity was normalised to total protein content of the protein extract and expressed as units of MPO activity/mg protein.

### Cord blood leukocyte count

Cord blood was collected before lamb euthanasia and analysed for neutrophils, lymphocytes and monocytes counts using an automated cell analyser (VetScanHM5, Abaxis, CA, USA).

### RNA isolation, reverse transcription and quantitative PCR

RNA purification, reverse transcription and quantitative PCR were performed as described by Song et al [20]. Diaphragm mRNA expression was measured to evaluate changes in cytokine genes (*IL-1β* and *IL-6*), proteolytic genes (muscle RING-finger protein-1 (*MuRF1*) and Muscle Atrophy F-Box (*MAFbx*) and anti-oxidant genes (Superoxide dismutase 1 (*SOD1*), Glutathione peroxidase 1 (*GPX1*) and *Catalase*). The fluorescence signal of samples was normalised against the average of 18S RNA and GAPDH. The 2^-ΔΔCT^ method [22] was used to calculate relative mRNA expression levels and presented as fold increase relative to controls.

### Biochemical analysis of oxidative stress and proteolysis

The activities of reduced (glutathione, GSH) and oxidised (glutathione disulphide, GSSG) glutathione in the diaphragm were measured using glutathione fluorescent detection kit (DetectX K006-F1, Arbor Assays, MI, USA) and expressed as GSH:GSSG ratio.

The 20 S proteasome levels in the diaphragm were measured fluorometrically in total protein extracts using an assay kit (BML-AK740 assay kit, Enzo Life Sciences, NY, USA). The specific activity of the proteasome was calculated according to kit instructions and normalised against total protein concentration.

The protein carbonyl content in diaphragm was measured using a commercially available protein carbonyl colorimetric kit (Cayman, Ann Arbor, MI, USA).

### Data analysis

Data are presented as mean (SEM) or median (range). Statistical analyses were performed using Sigmaplot (version 12.5, Systat Software Inc, USA). Differences among multiple groups were assessed using one-way ANOVA with post hoc analysis using Tukey honestly significant difference (HSD) test. Nonparametric data were examined using ANOVA on ranks. Statistical significance was accepted at p<0.05.

## Results

### Physiological variables at birth

Descriptive characteristics for each group are presented in Table 1. There were no significant differences in gestational age at birth, body weight or optimal muscle length between any of the groups.

**Table 1:** Lamb descriptive data and measures of diaphragm contractile function.

	Sal/Sal(n = 7)	Sal/LPS(n = 7)	rhIL-1ra/LPS(n = 8)	rhIL-1ra/Sal(n = 8)
Gestational age (d)	122.5 ± 0.2	121.7 ± 0.5	122.0 ± 0.3	121.8 ± 0.3
Body weight (kg)	2.7 ± 0.1	2.9 ± 0.1	2.8 ± 0.1	3.0 ± 0.0
L_0_ (mm)	28.6 ± 0.9	28.8 ± 0.9	29.4 ± 0.6	28.7 ± 1.0
TTP (s)	0.21 ± 0.02	0.28 ± 0.02	0.30 ± 0.02	0.26 ± 0.02
1/2 RT (s)	0.29 ± 0.01	0.28 ± 0.03	0.29 ± 0.01	0.26 ± 0.01
Max df/dt (g/s)	653 ± 46	573 ± 62	779 ± 41^#^	727 ± 35
TTP/P_t_ (s/N.cm^-2^)	0.023 ± 0.003	0.045 ± 0.004*	0.035 ± 0.003	0.026 ± 0.002
Twitch/Tetanus ratio	0.60 ± 0.02	0.63 ± 0.01	0.55 ± 0.02	0.64 ± 0.01
Fatigue index (FI)	0.58 ± 0.02	0.59 ± 0.02	0.57 ± 0.02	0.60 ± 0.02

L_0_—optimal muscle length; TTP—time to peak and 1/2 RT—half relaxation time of twitch contraction; df/dt—rate of force development; (FI index; lower value indicates increased fatigability). Values are mean ± sem.

* **—**significantly different to Sal/Sal (p<0.05).

^#^—significantly different to Sal/LPS (p<0.05)

### Diaphragm contractile function

**Fig 1:**
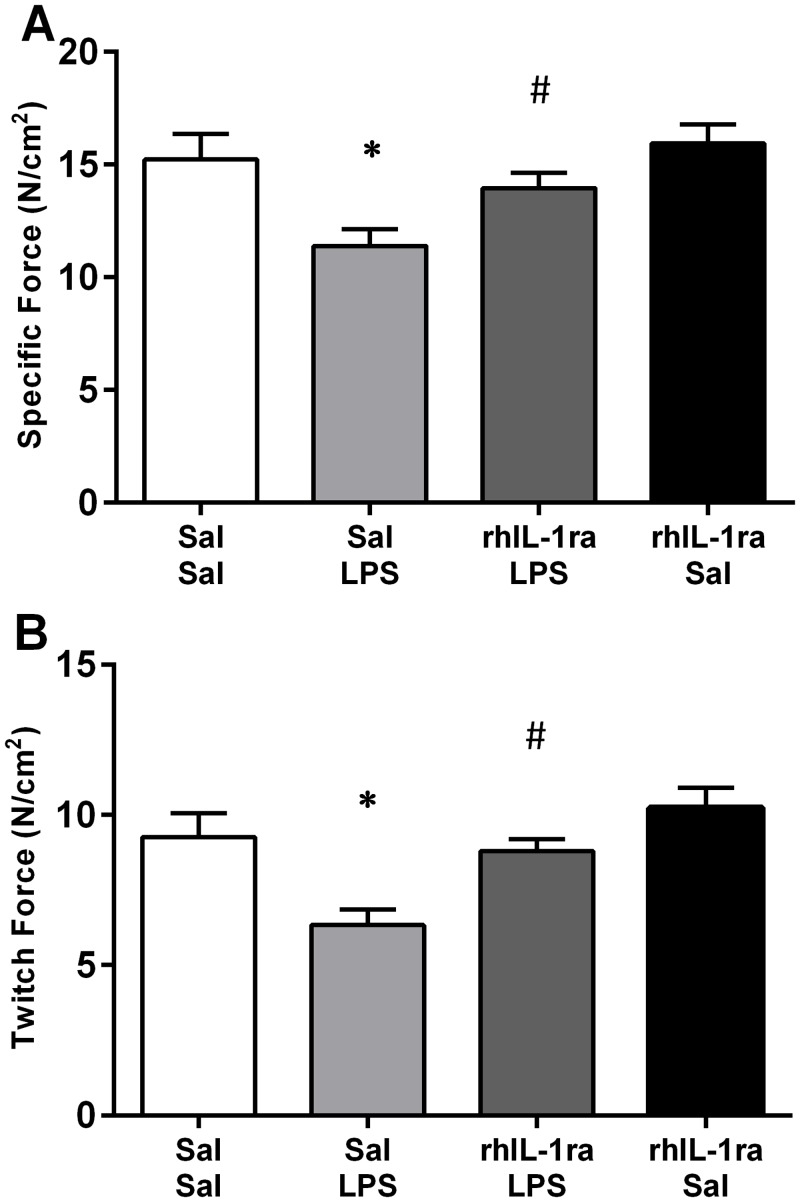
Fetal diaphragm contractile properties. Maximum specific force (A); and peak twitch specific force (B) for Sal/Sal (n = 7), Sal/LPS (n = 7), rhIL-1ra/LPS (n = 8) and rhIL-1ra/Sal (n = 8) exposure lambs. Values are mean ± SEM. * p<0.05 compared to Sal/Sal; ^#^ p<0.05 compared to Sal/LPS. Samples sizes are the same for subsequent figures.

Intra-amniotic (IA) LPS exposure two days prior to delivery significantly impaired diaphragm contractile function in preterm fetal lambs (Fig 1). Maximal specific force (P_0_) and twitch force (P_t_) in Sal/LPS lambs were 25% and 31% lower, respectively, compared to Sal/Sal control lambs. RhIL-1ra treatment three hours prior to IA LPS injection prevented the LPS induced decrease in P_0_ and P_t_. P_0_ and P_t_ in the rhIL-1ra/LPS group were not significantly different to Sal/Sal, but were significantly greater than Sal/LPS lambs (p = 0.044; p = 0.009, respectively). The rhIL-1ra treatment alone did not alter diaphragm contractile function as P_0_ and P_t_ were not significantly different between Sal/Sal and rhIL-1ra/Sal lambs.

The maximum rate of force development (df/dt) for twitch contractions was significantly greater for rhIL-1ra/LPS compared to Sal/LPS lambs (p<0.05). Although there were no significant differences in TTP between any groups (Table 1), when TTP was normalised to P_t_ (to account for differences in the amplitude of twitch contractions) the normalised TTP values were significantly higher in Sal/LPS lambs compared to Sal/Sal lambs (p<0.001; Table 1). Together these data reflect a relative slowing of the twitch contraction time in LPS exposed lambs which were attenuated by rhIL-1ra treatment. Other physiological contractile parameters (1/2 RT, twitch/tetanus ratio, and FI) were not significantly different between groups (Table 1).

### Systemic inflammation

**Fig 2:**
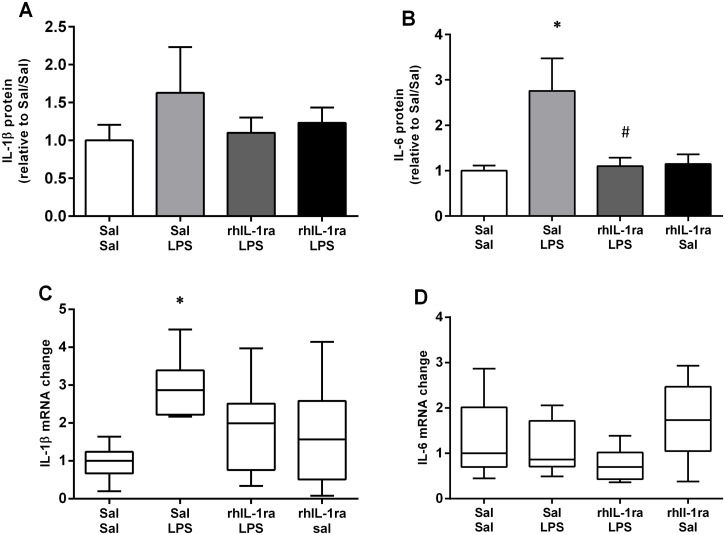
Systemic and diaphragm cytokine responses. Plasma IL-1β (A) and IL-6 (B) protein content. Values are mean ± SEM. Diaphragm IL-1β (C) and IL-6 (D) mRNA expression. Values are median (with 10^th^ and 90^th^ centiles). * p<0.05 compared to Sal/Sal; ^#^ p<0.05 compared to Sal/LPS.

There were no significant differences in the plasma IL-1β concentration between any of the experimental groups (Fig 2A). However, plasma IL-6 protein levels in the Sal/LPS group were significantly higher compared to the Sal/Sal group (p = 0.019) reflecting a systemic inflammatory response to IA LPS (Fig 2B). RhIL-1ra treatment prior to IA LPS injection inhibited the fetal systemic inflammatory response. Plasma IL-6 protein levels in the rhIL-1ra/LPS group were not significantly different to the Sal/Sal controls, but were significantly lower than in the Sal/LPS group (p = 0.023). Total white blood cell counts from cord blood were not significantly different between groups (Table 2).

**Table 2:** Cord blood leukocytes counts.

Group	Neutrophils(x 10^9^/L)	Monocytes (x 10^9^/L)	Lymphocytes (x 10^9^/L)
Sal/Sal	0.10 ± 0.02	0.019 ± 0.002	3.58 ± 0.03
Sal/LPS	0.24 ± 0.11	0.014 ± 0.002	2.41 ± 0.04
rhIL-1ra/LPS	0.24 ± 0.09	0.013 ± 0.002	2.13 ± 0.27
rhIL-1ra/Sal	0.15 ± 0.06	0.018 ± 0.003	3.48 ± 0.52

Values are mean ± sem

### Diaphragmatic inflammation

**Fig 3:**
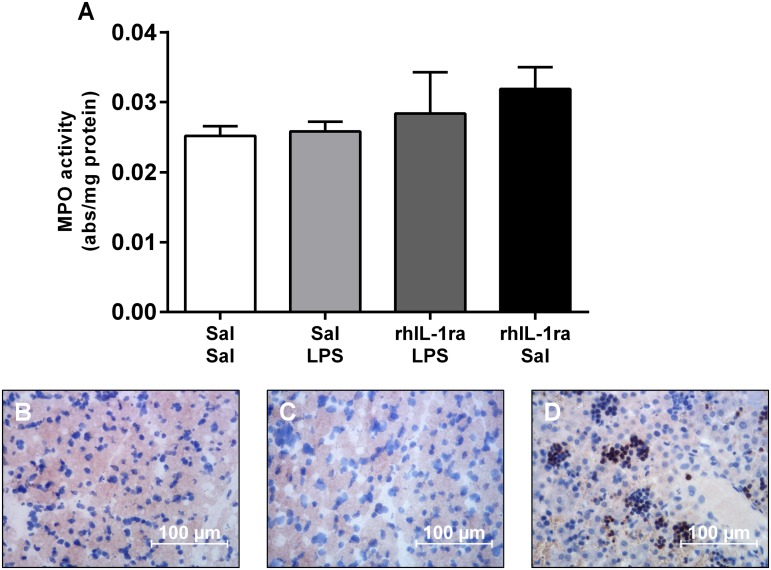
Diaphragm myeloperoxidase activity. MPO assay (A) (values are mean ± SEM) and images of MPO positive neutrophils in diaphragm cryosections from Sal/Sal (B) and Sal/LPS (C) lambs. Fetal liver (Sal/Sal; D) sections were used as positive control. MPO positive cells are stained brown. Magnification 400x, scale bars = 100 μm.

Diaphragm IL-1β mRNA expression was significantly higher in the Sal/LPS group compared to the Sal/Sal control group (p<0.05) (Fig 2C). Again, pre-treatment with rhIL-1ra inhibited the local diaphragmatic inflammatory response. Diaphragm IL-1β mRNA levels in the rhIL-1ra/LPS and rhIL-1ra/Sal groups were not significantly different to the Sal/Sal controls. There were no significant differences in the diaphragm IL-6 mRNA levels between any groups (Fig 2D). Histological and biochemical analyses of MPO revealed no significant difference in the number of inflammatory cells in the diaphragm after IA LPS exposure (Fig 3).

### Diaphragm atrophy gene expression and 20 S proteasome activity

**Fig 4:**
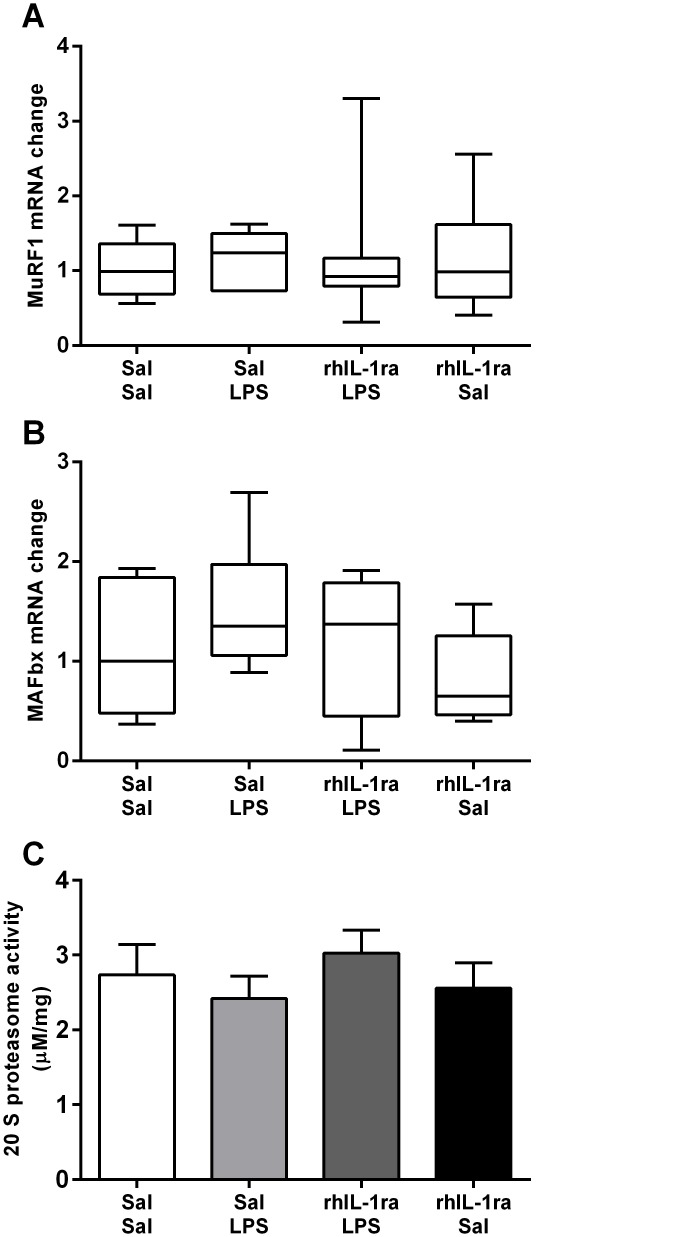
Atrophy related signalling in the diaphragm. Atrophy gene MuRF1 (A) and MAFbx (B) mRNA expression in diaphragm. Values are median (with 10^th^ and 90^th^ centiles). 20 S proteasome activity (C) normalised against total protein concentration. Values are mean ± SEM.

There were no significant differences in mRNA expression of muscle atrophy genes *MuRF1* and *MAFbx* (Fig 4A and 4B) or in 20 S proteasome activities (Fig 4C) between groups.

### Oxidative stress

**Fig 5:**
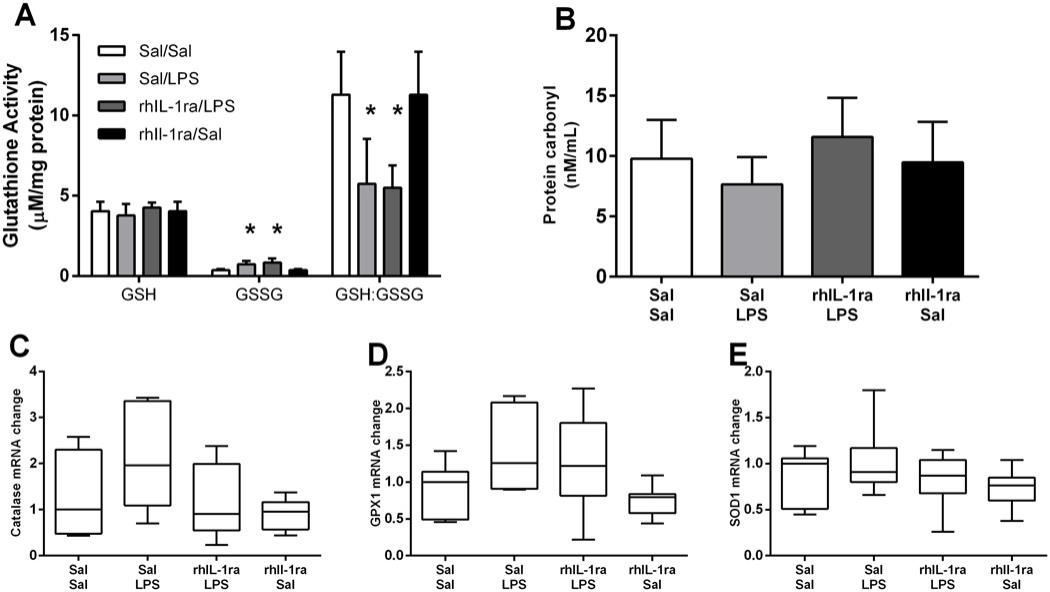
Oxidative stress in the diaphragm. Free and oxidised glutathione activity and GSH:GSSG ratio (A). Values are mean ± SEM. Protein carbonyl content (B). Values are mean ± SEM. Antioxidant genes *catalase* (C), *GPX1* (D), *SOD1* (E) mRNA expression in the diaphragm. Values are median (with 10^th^ and 90^th^ centiles). * p<0.05 compared with Sal/Sal.

IA LPS exposure caused oxidative stress in the diaphragm as reflected by a significantly higher oxidised glutathione (GSSG) level (p = 0.003) and consequently, a significantly lower GSH:GSSG ratio (p = 0.002) in Sal/LPS compared to Sal/Sal lambs (Fig 5A). In contrast to the inflammatory response, prior treatment with rhIL-1ra did not prevent the LPS induced increase in oxidative stress. GSSG was also significantly higher (p<0.001) and GSH:GSSG significantly lower (p = 0.004) in rhIL-1ra/LPS lambs compared to control lambs. There was no significant difference in GSSG between Sal/Sal and rhIL-1ra/Sal groups. Furthermore, there were no significant differences in protein carbonyl levels or mRNA expression of antioxidative genes (*catalase*, *GPX1*, *SOD1*) between groups (Fig 5B–5E).

## Discussion

Chorioamnionitis is associated with increased IL-1 levels in the amniotic fluid and IL-1 is the major contributor to lung proinflammatory activity and injury. IA LPS induced chorioamnionitis causes diaphragm muscle weakness [20]. We show that blocking IL-1 signalling via IA rhIL-1ra treatment ameliorates the diaphragm muscle weakness in preterm lambs. Blocking IL-1 also attenuates the LPS induced increase in systemic IL-6 levels and diaphragm IL-1β mRNA expression. These findings suggest that rhIL-1ra treatment protects against IA LPS induced diaphragm dysfunction by blocking the systemic and local inflammatory responses to *in utero* infection.

Previous animal studies show IL-1 pathway inhibition ameliorates inflammation related respiratory dysfunction. Pre-treatment with rhIL-1ra reduces the IL-1 induced damage to alveolar epithelial cells in a rat model of ventilator induced lung-injury [23]. Similarly, deletion of the IL-1 receptor type 1 (*IL-1R1*) gene in mice attenuates the pulmonary inflammatory response to aerosolised LPS [24] suggesting IL-1 signalling has an important role in lung inflammation and injury. Furthermore, blocking IL-1 signalling using IA rhIL-1ra injections reduces the pulmonary and systemic inflammation induced by IA exposure to LPS in preterm lambs [1]. Importantly, we show that the proinflammatory cytokine IL-1 is also implicated in IA LPS induced diaphragm dysfunction in preterm lambs.

Diaphragmatic weakness leading to acute respiratory failure is associated with increased expression of pro-inflammatory cytokines resulting from a systemic inflammatory response syndrome [14,15,25]. Cytokine levels in amniotic fluid and fetal cord blood increase in response to chorioamnionitis in both clinical and animal studies [1,21,26,27]. Monocyte chemotactic protein-1 (MCP-1) is a leukocyte chemoattractant and key regulator of the cytokine response to inflammation. Inhibition of MCP-1 with antibody neutralisation prevents diaphragm weakness in endotoxin treated mice [14] which suggests a coordinated cytokine response is critical in the development of inflammation related respiratory disorders.

We measured systemic and local (diaphragm) cytokine expression two days after IA LPS exposure. At this time plasma IL-6 protein level and diaphragm IL-1β mRNA expression are significantly elevated and blocking IL-1 signalling with rhIL-1ra prevented the increase in systemic and diaphragm inflammatory markers. The time-course of cytokine release initiated by IA LPS exposure suggests that IL-1β secretion occurs rapidly at the chorion/amnion [28,29] and precedes the release of secondary cytokines including IL-6. Numerous studies show that activating the IL-1 pathway stimulates IL-6 production in cultured mouse skeletal muscle cells [30], human lung fibroblast [31], endothelial cells [32] and neutrophils [33]. Although we could not measure cytokine levels at earlier time points in this study, we propose that the increase in systemic IL-6 is regulated by initial IL-1β secretion at the site of LPS exposure and leads to the induction of cytokine expression in the diaphragm. Previously we showed that local and systemic inflammatory responses to IA LPS are resolved within seven days, reflecting a progressive change in cytokine expression after LPS exposure [14]. These observations are consistent with the time course of cytokine expression characterised by Kallapur et al following IA LPS injection in preterm sheep [28].

Proinflammatory cytokines can impair contractile function by disrupting excitation-contraction coupling [13] and reducing muscle mass [34] via atrophy related signalling. Our previous study in preterm lambs [20] showed that IA LPS (10 mg) exposure initiated a complex response, characterised by an early (2 d) increase in pro-inflammatory cytokine expression and 20S proteasome activity, followed by a significant decrease in protein synthesis activity and atrophy related gene expression at 7 d after the initial LPS exposure. In the current study, using a lower dose of LPS (4mg) we show that 20S proteasome enzyme activity and *MURF1* and *MAFbx* gene expression after IA LPS exposure were not different to control levels suggesting that the diaphragm weakness that we observed at 2 d after a low dose IA LPS exposure was not due to muscle wasting. However, because sampling at two days after LPS exposure may have failed to identify a transient increase in gene expression, and a lack of ovine specific antibodies prevented us from measuring MuRF1 or MAFbx protein expression in these samples, we cannot exclude the possible involvement of atrophy signalling in the current study. We believe this is unlikely for two reasons: Firstly, our previous study showed that at two days after LPS exposure there was no significant difference in the proportion or cross-sectional area of MHCI or MHCII positive fibres in the diaphragm [20]. Secondly, the diaphragm weakness that we observed in this study was reflected by a significantly lower maximum specific force in the 2 d LPS group compared to controls. Because the specific force is a measure of force production relative to the amount of muscle tissue, it is unlikely to be affected by muscle wasting. Therefore, it is likely that the initial inflammation related diaphragm weakness that we observed was mediated by alterations to excitation-contraction coupling [13,15].

Our analysis of the time course for twitch contractions suggest that IA LPS exposure slows the twitch contraction times and this slowed contraction is prevented by blocking the IL-1 pathway. These findings are consistent with LPS-induced alteration to the calcium release mechanism. This proposed mechanism is supported by the IL-1β associated decrease in sarcoplasmic reticulum Ca^2+^ release, achieved by altering L-type Ca^2+^ channel [35] and ryanodine receptor [36] function in cardiac muscle. In skeletal muscle, IL-1α (that binds to the same receptor as IL-1β) directly inhibits sarcoplasmic reticulum Ca^2+^ release by inhibiting ryanodine receptor activation [37].

Interestingly, rhIL-1ra treatment does not protect the diaphragm against LPS induced oxidative stress. IA LPS was associated with elevated GSSG levels and consequently, a reduced GSH:GSSG ratio, and this response was not altered by rhIL-1ra treatment. These findings suggest that the LPS induced preterm ovine diaphragm dysfunction is not mediated by oxidative stress. However, it is worth noting that there were no changes in other measures of oxidative stress (protein carbonyl content, or mRNA expression of antioxidant genes *SOD1*, *GPX1* or *Catalase*) after a two day IA LPS exposure: therefore, the overall level of oxidative stress may be relatively low at this time point. Further, our observation that MPO activity and MPO positive inflammatory cells in the diaphragm were unaltered two days after LPS exposure is consistent with previous reports showing LPS induced diaphragm weakness can occur without changes in the intramuscular levels of neutrophils or macrophages [14]. The modest changes in monocytes and oxidative stress markers may reflect the lower dose of LPS used here (4 mg) compared to previous studies (10–20 mg) [1,20,28]. Although an IA LPS dose response study showed that 4 mg and 10 mg IA endotoxin caused similar inflammation in the lung and chorioamnion and lung maturation [29], it is possible that the inflammatory response is somewhat weaker in the more distal diaphragm muscle. While IL-1 signalling is an important contributor to LPS induced inflammation, other pathways downstream of toll-like receptor 4 activation also contribute to fetal inflammation and oxidative stress [1].

Although our results indicate that IA LPS exposure did not alter the fatigue resistance of the preterm diaphragm, extrapolation of these results to the clinical setting should be made with caution. The *in vitro* fatigue protocol used in this study involved maximal isometric contractions of isolated diaphragm fibres. Although this technique may be adequate for evaluating the decrease in maximum force producing capacity over time, it is unlikely to accurately reflect the *in vivo* function in which the diaphragm is activated at submaximal levels and is required to contract against a compliant rib cage. Respiratory fatigue in the clinical setting reflects the balance between the work performed by the diaphragm during breathing, and the functional capacity of the diaphragm. Due to the significant (25%) reduction in the force producing capacity, the diaphragm of LPS exposed animals is more likely to be operating closer to maximal functional capacity and therefore any level of fatigue may result in the development of insufficient spontaneous respiratory effort and respiratory failure.

In conclusion, IA LPS exposure causes diaphragm weakness in preterm lambs and blockade of IL-1 signalling protects the diaphragm from inflammation induced contractile dysfunction. We suggest that the IL-1 pathway is implicated in diaphragm weakness following LPS induced chorioamnionitis and IL-1 may directly affect excitation-contraction coupling. Diaphragmatic dysfunction due to immature muscle function, increased work load, inflammatory insult and/or fatigue may contribute to postnatal respiratory failure in preterm infants. Therefore, IL-1 may be an attractive therapeutic target in chorioamnionitis induced diaphragm dysfunction.
